# Microstructure and Properties of Densified Gd_2_O_3_ Bulk

**DOI:** 10.3390/ma15217793

**Published:** 2022-11-04

**Authors:** Pei-Hu Gao, Can Jin, Sheng-Cong Zeng, Rui-Guang Xie, Bo Zhang, Bai-Yang Chen, Zhong Yang, Yong-Chun Guo, Min-Xian Liang, Jian-Ping Li, Li-Na Zhang, Zhi-Yi Yan, Lu Jia, Dan Zhao

**Affiliations:** 1 School of Materials and Chemical Engineering, Xi’an Technological University, Xi’an 710021, China; 2 Shaanxi Province Engineering Research Centre of Aluminum, Magnesium Light Alloy and Composites, Xi’an 710021, China; 3 Xi’an Aerospace Composite Research Institute, Xi’an 710025, China; 4 Shanxi Diesel Engine Co., Ltd., Datong 035600, China

**Keywords:** Gd_2_O_3_ bulks, sinter, thermal conductivity, hardness, modulus, fracture toughness

## Abstract

In this work, Gd_2_O_3_ bulks were sintered at temperatures ranging from 1400 °C to 1600 °C for times from 6 h to 24 h, and their microstructure and properties were studied for a wider application of materials in thermal barrier coatings. The densification of the Gd_2_O_3_ bulk reached 96.16% when it was sintered at 1600 °C for 24 h. The elastic modulus, hardness, fracture toughness and thermal conductivity of the bulks all increased with the rise in sintering temperature and extension of sintering time, while the coefficient of thermal expansion decreased. When the Gd_2_O_3_ bulk was sintered at 1600 °C for 24 h, it had the greatest elastic modulus, hardness, fracture toughness and thermal conductivity of 201.15 GPa, 9.13 GPa, 15.03 MPa·m^0.5^ and 2.75 W/(m·k) (at 1100 °C), respectively, as well as the smallest thermal expansion coefficients of 6.69 × 10^−6^/°C (at 1100 °C).

## 1. Introduction

Thermal barrier coatings (TBCs) have been commonly applied to hot-end components in progressive gas turbines and aero engines to enhance engine dependability, durability as well as efficiency [1,2,3]. TBCs can effectively isolate the touch of the high-temperature working agent with the metal substrate, thus reducing the components’ surface temperature and weakening the heat transfer efficiency, which ultimately plays a role in protecting the metal substrate [4,5,6]. The top ceramic layer is essential in the thermal insulation of the thermal barrier coating, which is required to possess a high melting point, low heat exchange rate, stable crystal structure as well as good anti-sintering abilities. ZrO_2_ is a commonly used material in TBCs for its high melting point (2680 °C), good anti-oxidation activity, stable chemical activity, high shock resistance and a coefficient of thermal expansion (CTE) close to metals (8~10.4 × 10^−6^/°C). However, when it is used at high temperature, the phase structure will change, resulting in a change in volume, and the stress in the coating increases, which will cause the initiation of cracking, leading to the failure of the ceramic layer [7,8,9,10,11,12,13].

In Liu’s work, it was proposed that by adjusting the composition, thermal barrier coatings can be prepared with lower thermal conductivity and better high temperature performances than the commonly used ceramics materials [14]. The structure of zirconia TBCs can be modified by rare-earth oxide co-doped with trivalent or pentavalent compounds [15]. In lots of stabilizers of ZrO_2_, Y_2_O_3_ is seen as the most suitable stabilizer when its doping amount lies from 6 to 12 wt%. Specially, 8 mol-YSZ has a single stabilized cubic structure with a thermal conductivity of 2.3 and 1.85 W/(m·k) at room temperature and 1000 °C, respectively [16]. Conversely, once the serving temperature is higher than 1200 °C, ZrO_2_ will be sintered quickly accompanied with phase changes. Rare-earth oxides become stable once they are oxidized because rare earth elements have a strong oxygen affinity [17,18]. The melting point of Gd_2_O_3_ is 2350 °C. Meanwhile, it possesses stable chemical activity, good anti-oxidation activity and good shock resistance, which makes it suited to serving in extreme environments [19,20,21,22]. The structure of TBCs can be modified and become stable via interaction with oxide co-doped yttrium oxide-stabilized zirconia ceramic materials. Moreover, Gd possesses a powerful ability of adsorbing rest elements, good chemical stability, and can be dissolved in ZrO_2_ cell [23,24,25]. Gao et al. [26] prepared quaternary GYYZO bulks with Gd_2_O_3_ and Yb_2_O_3_ added into 8YSZ. It was found that the addition of Gd_2_O_3_ reduced the thermal conductivity while improving the mechanical properties of the TBCs, with better comprehensive performance. Bobzin K et al. [27] prepared Yb_2_O_3_-Gd_2_O_3_ co-doped YSZ high-porosity TBCs by atmospheric plasma spraying (APS), and thermally cycled the product at 1150 °C to study the sintering effects on the coatings’ microstructure and properties. The Yb_2_O_3_-Gd_2_O_3_ co-doped YSZ coating has a relatively low thermal conductivity of 1.1 W/(m·K) (at 1100 °C). Zhang et al. [28] prepared Yb_2_O_3_ and Gd_2_O_3_ co-doped SrZrO_3_ system with good properties through a traditional solid-state reaction. The SZYG/YGZO composite ceramics with Yb_0.5_Zr_0.5_O_1.75_ and SZO phases possessed a thermal conductivity of 1.3 W/(m·K) (at 1000 °C), which was 40% less than the SZO ceramics’ at least. The CTE of the SZYG/YGZO composite ceramics reached 10.9 × 10^−6^K^−1^ (1250 °C). Meanwhile, the SZYG/YGZO composite ceramics’ fracture toughness was 30% higher than that of the SZO ceramic. Zheng et al. [29] deposited TBCs with Sm-doped Gd_2_Zr_2_O_7_ through EB-PVD (electron beam physical vapor deposition: EB-PVD). The coating exhibited high CTE and long thermal shock lifetimes at 1100 °C. The TGO’s thickness was about 15 μm after thermal shock tests. Therefore, the addition of Gd elements could evidently improve the performance of ZrO_2_ TBCs.

However, how does the added Gd_2_O_3_ enhance the material properties used in TBCs? This requires the properties of the pure Gd_2_O_3_, while there are few reports on the properties of Gd_2_O_3_ [19,20,21,22,23,25,26,27,28,29]. By consulting the relevant literature [19,21,22,29], it can be found that there are no detailed values for the thermal physical properties, including the thermal conductivity and thermal expansion coefficient, as well as mechanical properties, including elastic modulus, hardness and fracture toughness of the pure Gd_2_O_3_. Therefore, in this work, Gd_2_O_3_ bulks were made with which to study the microstructure, mechanical properties and thermal physical properties, which made the foundations for the wider application of materials in TBCs.

## 2. Experimental Materials and Procedures

### 2.1. Preparation of Gd_2_O_3_ Bulks

**Figure 1:**
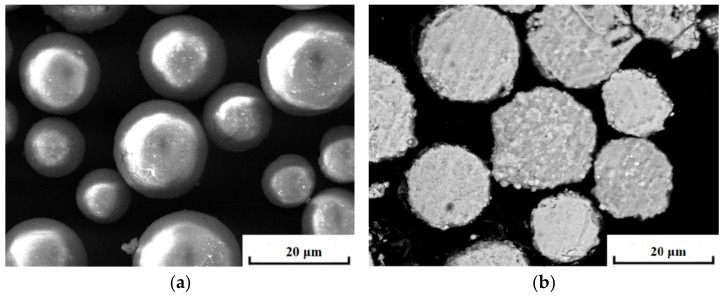
Globular morphology of Gd_2_O_3_ powders (**a**); Cross-sectional microstructure of Gd_2_O_3_ powders (**b**).

For the convenience of preparing the bulk, Gd_2_O_3_ powders were milled by ball milling with the type of NDL-04, which was made in Xianyang zunkai Co., Ltd., Xianyang, China. The ball milling rotation rate was controlled at 120 revolutions per minute. Table 1 showed the ball milling technology. During the milling of powders, the ball-to-powder ratio in weight was set as 10:1. Both the ball and jar consisted of agate in order to avoid contamination. The main component of agate was silicon dioxide with a Vickers hardness of 1213 ± 75. The powders were milled for 20 h. Sodium stearate was used as a wetting agent in one-percent solution during milling. The initial mean particle size of the Gd_2_O_3_ powder was about 100 µm. The ball-milled Gd_2_O_3_ powder’s mean size was 20 µm. The powders’ morphology and cross-sectional microstructure were shown in Figure 1. The powder had a globular shape. Then, the powders were cold-pressed into a green body in a size of φ13 mm × 3 mm by an isostatic press with the type of TYP-60T, which was made in Taiyuan Xinzuo Co., Ltd., Taiyuan, China. The compacting pressure was kept at 200 MPa for 5 min. The detailed cold compaction parameters were given out in Table 2. Then, the preforms were sintered at 1400 °C, 1500 °C, 1600 °C for 6 h to 24 h in an air ambient muffle furnace, respectively. The sintering temperature was increased from room temperature to 600 °C at a heating rate of 10 °C/min. Then, the samples were heated from 600 °C to 1400 °C, 1500 °C and 1600 °C at a heating rate of 3 °C/min, respectively. The cooling method chosen was furnace cooling after heating.

### 2.2. Microstructures and Phases

The original powders’ morphology and the sintered bulks’ microstructure were characterized through a scanning electron microscopy conducted with the type of VEGA II-XMU, which was made in TESCAN, Brno, Czech Republic. X-ray diffraction was conducted with the D8 Discover, which was made in Bruker AXS GmbH, Germany, and was applied to characterize the powders’ and the bulks’ phases with Kα radiation of cooper. X-ray scanning was carried out with a step of 0.02°. The scanning was applied with 2θ from 20° to 80°. The scanning speed was controlled at 2°/min.

### 2.3. Properties

The bulk’s density was tested according to the Archimedes drainage method. The bulk’s mass was weighted through a balance with the type of PTY-504, which was made in Funing Huazhu Instrument Co., Ltd., Funing, Jiangsu, China. The balance’s accuracy was 0.0001 g. The density could be calculated through Formula (1):

(1) $$\rho_{s}=\frac{m_{1}}{m_{1}-m_{2}}(\rho_{0}-\rho_{L})+\rho_{L}$$

where *ρ*_s_ indicated the sample’s density, *ρ*_0_ indicated the water’s density of 0.998 g/cm^3^, *ρ*_L_ indicated the air’s density of 1.2 × 10^−3^ g/cm^3^, m_1_ indicated the sample’s mass measured in air, and m_2_ indicated the sample’s mass measured in water. The mean value of 10 measurements per sample was used for experimental data.

A laser thermal conductivity meter with the type of DLF-1200, which was made in TA, New Castle, DE, USA, was applied to test the thermal conductivity according to specifications of the laser flash heating technique. The thermal conductivity was estimated according to Formula (2):

(2) $$\lambda =DC_{p}\rho$$

where λ indicated the thermal conductivity (W·m^−1^·K^−1^), D indicated the thermal diffusivity (m^2^·s^−1^), C_p_ indicated the specific heat (J·kg^−1^·K^−1^), and ρ indicated the sample’s density at room temperature (kg·m^−3^).

A thermal expansion meter (SDTA840, TA, New Castle, DE, USA) was applied to test the thermal expansion coefficient. The CTE was estimated through Formula (3):

(3) $$\alpha =\frac{\left(L_{T}-L_{0}\right)}{L_{0}\left(T-T_{0}\right)}$$

where α represented the material’s CTE, L_T_ and L_0_ represented the sample’s length at the temperature of T and T_0_, and T_0_ represented room temperature, respectively.

A nanomechanical testing system with the type of Hysitron TI Premier, which was made in Bruker, USA, was applied to measure modulus, hardness and fracture toughness. The used indenter in tests was a prismatic indenter. The force of 10 mN was loaded linearly in 5 s. The load of 10 mN was kept for 3 s. The load of 10 mN was unloaded linearly in 5 s. Radical cracks formed on the surface of the samples when the indentation test was used in a low-load model. All the formed cracks were radial cracks in this work. The crack length could be determined. The fracture toughness can be calculated through Formula (4) [29]:

(4) $$K_{IC}=1.073•\alpha •{\left(\frac{E}{H}\right)}^{1/2}•\left(\frac{P}{C^{3/2}}\right)$$

where P represented the maximum press in load, C represented the crack length, and α represented a correlation coefficient related to the indenter appearances; where 1.6 × 10^−2^ was adopted, E represented the elastic modulus, and H represented the hardness. The mean values of the hardness, elastic modulus and fracture toughness were adopted on the base of ten measured results.

## 3. Results

### 3.1. Microstructure

**Figure 2:**
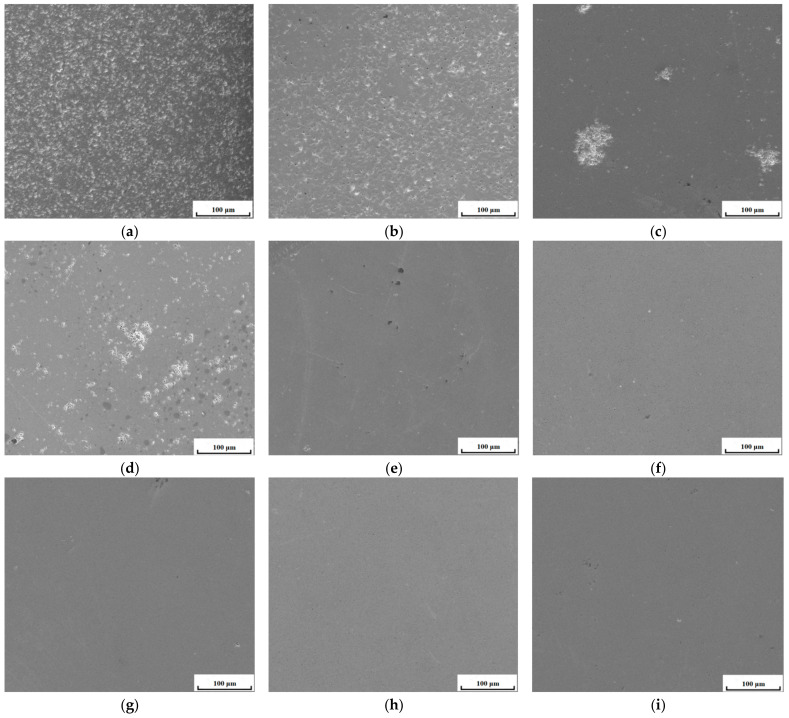
The Gd_2_O_3_ bulk’s microstructure, sintered at 1400 °C for 6 h (**a**), 12 h (**b**), and 24 h (**c**); the Gd_2_O_3_ bulk’s microstructure, sintered at 1500 °C for 6 h (**d**), 12 h (**e**), and 24 h (**f**); the Gd_2_O_3_ bulk’s microstructure, sintered at 1600 °C for 6 h (**g**), 12 h (**h**), and 24 h (**i**).

Figure 2 shows the Gd_2_O_3_ bulks’ microstructures as being sintered at 1400 °C, 1500 °C, 1600 °C, for 6 h to 24 h. The sintered Gd_2_O_3_ bulks became dense gradually when the sintering time was extended at 1400 °C. In Figure 2a, when the Gd_2_O_3_ bulk was sintered at 1400 °C for 6 h, there existed were a lot of pores, while the number of pores in the bulk material decreased significantly when the fritting times reached 12 h and 24 h, as shown in Figure 2b,c. The bulks’ pores dissolved gradually with the fritting and densification of Gd_2_O_3_. With the increase in the fritting temperature, the sintered Gd_2_O_3_ bulk became dense. In Figure 2d, when the Gd_2_O_3_ bulk was sintered at 1500 °C for 6 h, a lot of pores were also existed, while the number of pores in the bulk material decreased significantly when the fritting times reached 12 h and 24 h as shown in Figure 2e,f. As compared with the Gd_2_O_3_ sintered at 1400 °C, the number of pores was lower in the one sintered Gd_2_O_3_ at 1500 °C. The Gd_2_O_3_ bulk sintered at 1600 °C had almost no pores. It reached a state of complete densification. With the sintering time extension at 1600 °C, the pore numbers did not change in the sintered Gd_2_O_3_ bulk, as seen in Figure 2g–i, which was different to the densification of the sintered Gd_2_O_3_ bulk at 1400 °C and 1500 °C.

The bulks’ porosities were processed through the image processing method with ImageJ Software^@^. Table 3 shows the porosities of the bulks. With the fritting time extension, the bulks’ porosities decreased gradually. There was the lowest porosity in the bulk sintered for 24 h. Meanwhile, the sintered Gd_2_O_3_ bulk at 1600 °C had almost no pores, and was reaching a state of complete densification. With the fritting time extension at 1600 °C, the change of the pores was not obvious in the sintered Gd_2_O_3_ bulk.

### 3.2. Phases

**Figure 3:**
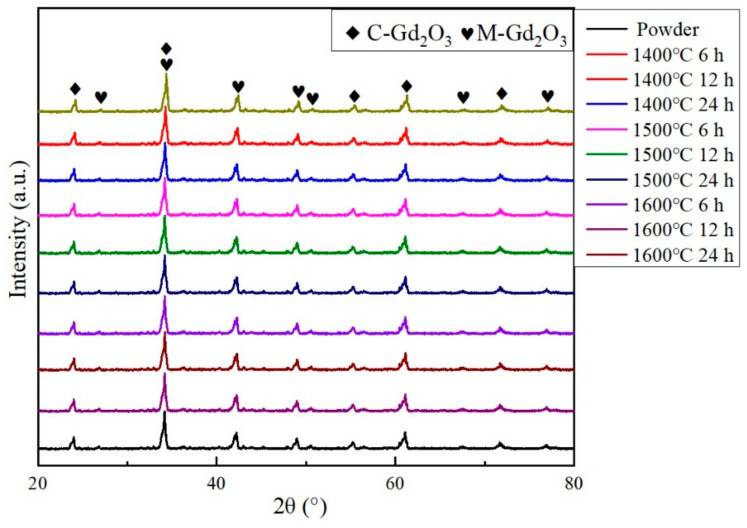
X-ray diffraction patterns of the Gd_2_O_3_ powder and the sintered bulks.

Figure 3 shows the X-ray diffraction patterns of the original powders and the sintered Gd_2_O_3_ bulks. On the bases of X-ray diffraction peaks identification, all of the peaks have been indicated. The Gd_2_O_3_ powder and sintered bulks consisted of both cubic and monoclinic structures. According to the Rietveld method, the ratios of cubic and monoclinic phases of Gd_2_O_3_ were 90.02% and 9.98%, respectively. Neither of the Gd_2_O_3_ powder and bulks were pure cubic or monoclinic, while the Gd_2_O_3_ powder and bulks were composed mainly of the cubic phase with about ten percent of monoclinic phase. The Gd_2_O_3_ bulks possessed the same phases as the original powder. The Gd_2_O_3_ bulk had no obvious phase transitions during sintering at 1400 °C, 1500 °C and 1600 °C. The characteristic peaks of the sintered Gd_2_O_3_ bulks were identical.

### 3.3. Densification

Table 4 shows the sintered Gd_2_O_3_ bulks’ real densities, tested according to the Archimedes drainage method. The sintered Gd_2_O_3_ bulk became dense gradually when the sintering temperature and the sintering time were increased. The density became larger and larger. The bulk had the maximum density of 7.394 g/cm^3^ when it was sintered at 1600 °C for 24 h.

The theoretical densities of the pure monoclinic and cubic Gd_2_O_3_ were 8.350 g/cm^3^ and 7.616 g/cm^3^, respectively. The Gd_2_O_3_ bulks with 9.98% monoclinic and 90.02% cubic phases had a theoretical density of 7.689 g/cm^3^ according to the contents of each phase. The sintered bulks’ densifications were determined according to the ratio of the real density to the theoretical one. The densifications of the Gd_2_O_3_ bulks sintered at different temperature for different times were given out in Table 5. With the rise in fritting temperature and the extension of fritting time, the sintered Gd_2_O_3_ bulk became dense, and the densification increased gradually. The Gd_2_O_3_ bulk had the maximum densification of 96.16% when it was sintered at 1600 °C for 24 h. The reported density of the Gd_2_O_3_ bulk was 7.407 g/cm^3^. The alumina was often added to Gd_2_O_3_ bulk to lower the sintering temperature and accelerate the sintering process. The alumina’s density was about 3.5 g/cm^3^. Therefore, the reported Gd_2_O_3_ bulk’s density was lower than the theoretical one.

### 3.4. Thermal Conductivity

**Figure 4:**
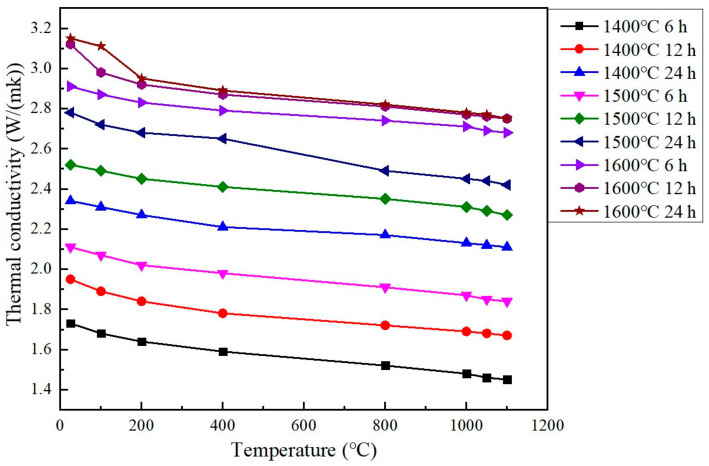
The sintered Gd_2_O_3_ bulks’ thermal conductivities.

As shown in Figure 4, the Gd_2_O_3_ bulks’ thermal conductivities were detected from room temperature to 1100 °C. With the extension of fritting time at a certain temperature, the bulk’s thermal conductivity increased gradually. The Gd_2_O_3_ bulk possessed the minimum thermal conductivity of 1.45 W/(m·k) (at 1100 °C) when it was sintered at 1400 °C for 6 h, which was much less than that of the one sintered at 1400 °C for 24 h, with its value of 2.11 W/(m·k) (at 1100 °C). The Gd_2_O_3_ bulk possessed the thermal conductivity of 1.84 W/(m·k) (at 1100 °C) when it was sintered at 1500 °C for 6 h, which was less than that of the one sintered at 1500 °C for 24 h with its value of 2.42 W/(m·k) (at 1100 °C). The Gd_2_O_3_ bulk sintered at 1600 °C for 6 h, 12 h, 24 h possessed the thermal conductivity of 2.68, 2.75 and 2.75 W/(m k) at 1100 °C, respectively. When the fritting temperature was 1600 °C, the thermal conductivity was almost unchanged with the extension of fritting time, which was attributed to the fully densified state of the Gd_2_O_3_ bulk sintered at 1600 °C. The densified Gd_2_O_3_ bulk’s thermal conductivity was 2.75 W/(m·k) (at 1100 °C). With the increase in the sintering temperature, the pores or voids inside or among the Gd_2_O_3_ powders disappeared gradually. The motionless air in the pores or voids was a poor conductor of heat, with low thermal conductivity. With the decrease in the pores and voids during sintering, the sintered bulk’s Gd_2_O_3_ thermal conductivity was increased gradually.

### 3.5. Thermal Expansion Coefficient

**Figure 5:**
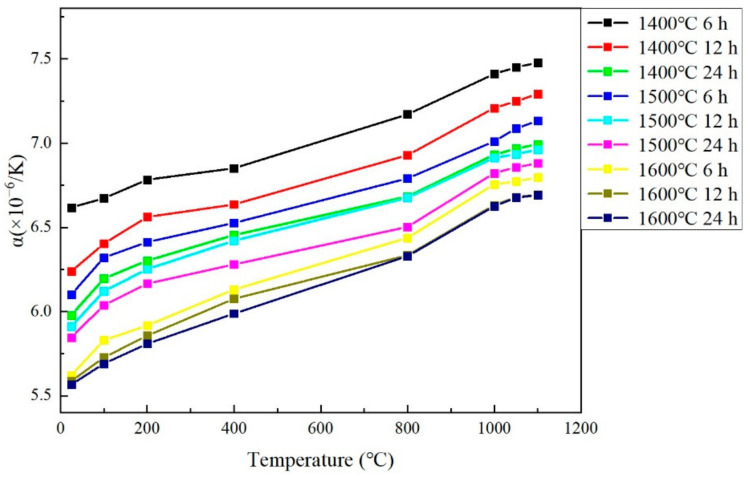
The sintered Gd_2_O_3_ bulks’ CTEs.

As shown in Figure 5, the Gd_2_O_3_ bulks’ CTEs were detected from room temperature to 1100 °C. Generally, all of the sintered bulks’ CTEs increased when the working temperature elevated. The bulks’ CTEs decreased gradually with the rise in sintering temperatures and extension of times. The sintered Gd_2_O_3_ bulk possessed the minimum CTE of 6.69 × 10^−6^/°C (at 1100 °C) when it was sintered at 1600 °C for 24 h, which was 10.6% less than that of the one sintered at 1400 °C for 6 h with its CTE of 7.48 × 10^−6^/°C (at 1100 °C). The CTEs of the bulks sintered at 1600 °C were almost unchanged, which was attributed to the fully densified state of the Gd_2_O_3_ bulk sintered at 1600 °C. At 1100 °C, the densified Gd_2_O_3_ bulk possessed a CTE of 6.69 × 10^−6^/°C. The increase in the bulk density with the increase in sintering temperature could be attributed to the decrease in pores or voids inside or among the Gd_2_O_3_ powders. With the densification process, the thermal expansion space became smaller, which restricted the thermal expansion and led to a low thermal expansion coefficient. With the decrease in the pores and voids during sintering, the sintered bulk’s thermal expansion coefficient decreased gradually.

### 3.6. Mechanical Properties

**Figure 6:**
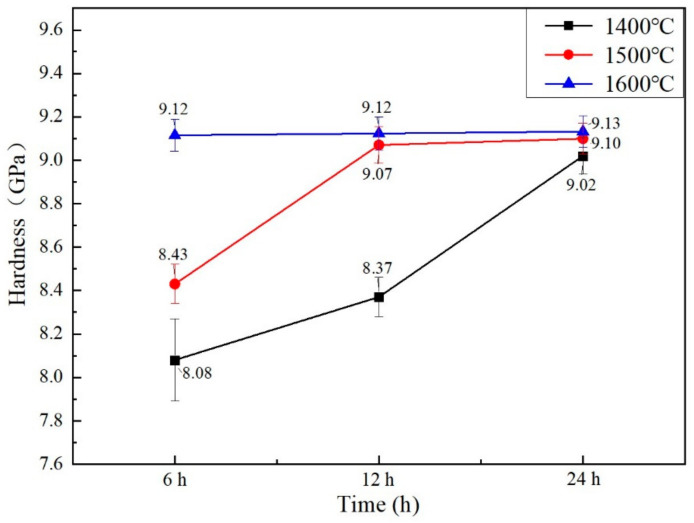
The sintered Gd_2_O_3_ bulks’ hardnesses.

Figure 6 shows the hardnesses of the bulks sintered at 1400 °C, 1500 °C, 1600 °C for 6 h, 12 h, and 24 h. With the rise in the sintering temperatures and extension of the fritting times, the sintered Gd_2_O_3_ bulks’ hardnesses were increased. The Gd_2_O_3_ bulk had the minimum hardness of 8.08 GPa when it was sintered at 1400 °C for 6 h, which was 10.4% less than that of the sample sintered at 1400 °C for 24 h, with its value of 9.02 GPa. The Gd_2_O_3_ bulk possessed the hardness of 8.43 GPa when it was sintered at 1500 °C for 6 h, which was 7.4% less than that of the one sintered at 1500 °C for 24 h, with its value of 9.10 GPa. The Gd_2_O_3_ bulks possessed the hardnesses of 9.12 GPa, 9.13 GPa and 9.13 GPa, respectively, when they were sintered at 1600 °C for 6 h, 12 h and 24 h. The hardness was almost unchanged, which was attributed to the fully densified state of the Gd_2_O_3_ bulk sintered at 1600 °C. The densified Gd_2_O_3_ bulk with the hardness of 9.13 GPa. With the extension of fritting times, the bulks’ densifications reached 99.82%, which was near the full densification and could indicate high hardness.

**Figure 7:**
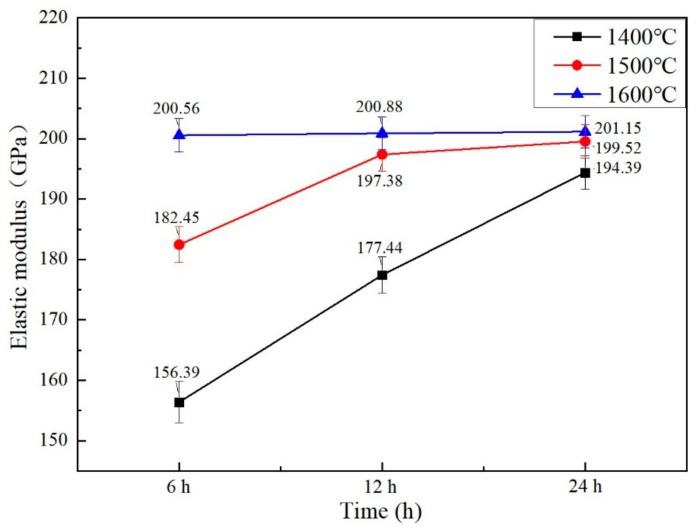
The sintered Gd_2_O_3_ bulks’ elastic moduli.

As Figure 7 shown, the bulks’ elastic moduli were measured when they were sintered at 1400 °C, 1500 °C, 1600 °C for 6 h to 24 h. When the sintering temperature was raised and the sintering time was extended, the bulk’s elastic modulus increased. The Gd_2_O_3_ bulk possessed the minimum elastic modulus of 156.39 GPa when it was sintered at 1400 °C for 6 h, which was 19.5% less than that of the one sintered at 1400 °C for 24 h with its value of 194.39 GPa. The Gd_2_O_3_ bulk possessed the elastic modulus of 182.45 GPa when it was sintered at 1500 °C for 6 h, which was 8.5% less than that of the one sintered at 1500 °C for 24 h, with its value of 199.52 GPa. The Gd_2_O_3_ bulks possessed the elastic modulo of 200.56 GPa, 200.88 GPa, 201.15 GPa, respectively, when they were sintered at 1600 °C for 6 h, 12 h, 24 h. The elastic modulus was almost unchanged, which was attributed to the fully densified state of the Gd_2_O_3_ bulk sintered at 1600 °C. The densified Gd_2_O_3_ bulk’s elastic modulus was 201.15 GPa.

**Figure 8:**
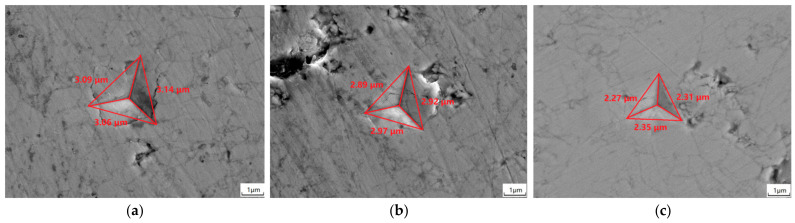

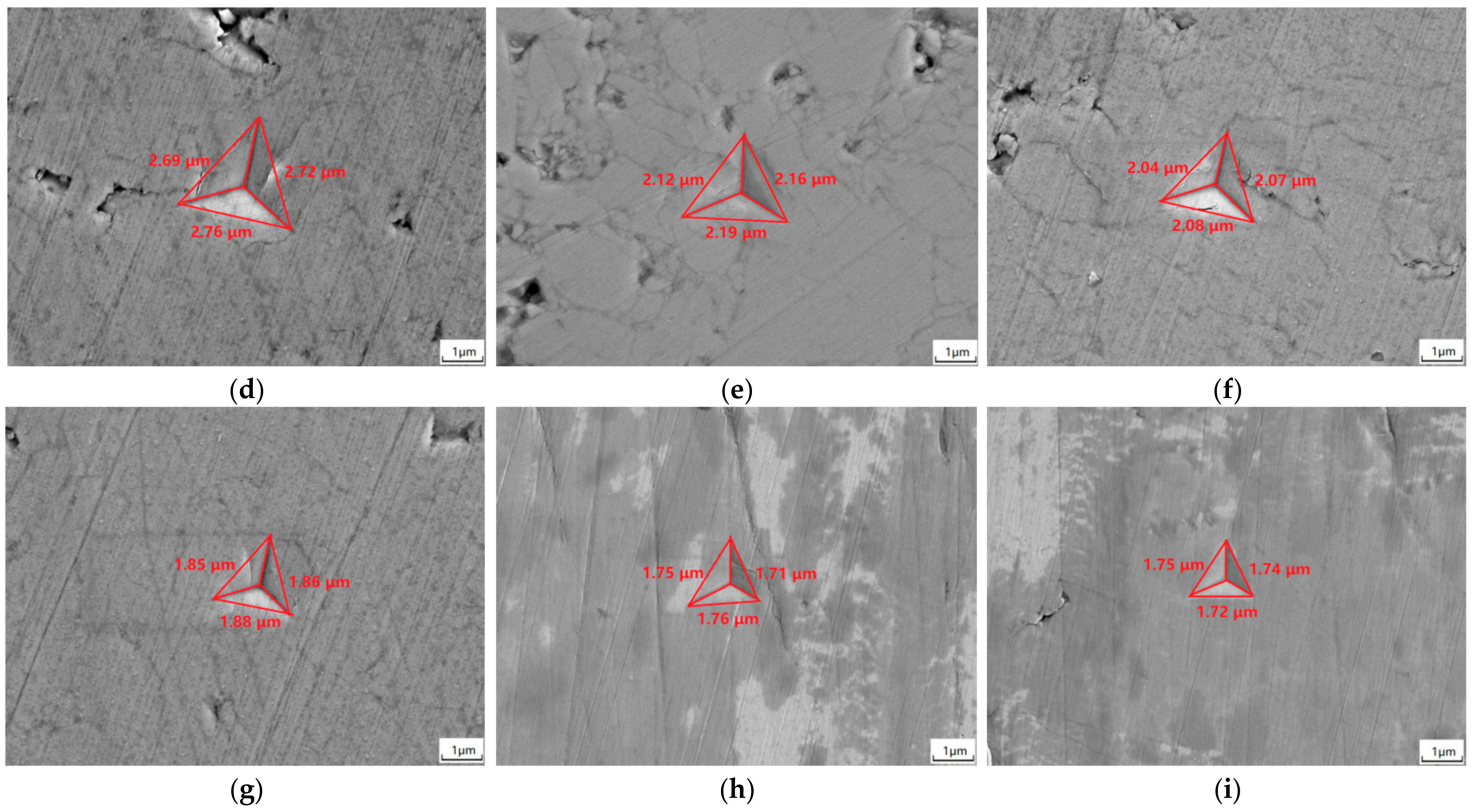
Indentations of the Gd_2_O_3_ bulks sintered at 1400 °C for 6 h (**a**), 12 h (**b**), and 24 h (**c**); indentations of the Gd_2_O_3_ bulks sintered at 1500 °C for 6 h (**d**), 12 h (**e**), and 24 h (**f**); indentations of the Gd_2_O_3_ bulks sintered at 1600 °C for 6 h (**g**), 12 h (**h**), and 24 h (**i**).

As Figure 8 shown, the nanoindentations with cracks in the Gd_2_O_3_ bulks were labeled when the bulks were sintered at 1400 °C, 1500 °C, 1600 °C for 6 to 24 h. Table 6 shows the bulks’ fracture toughnesses (K_IC_) estimated on the basis of Formula (4). When the sintering time extended, the sintered Gd_2_O_3_ bulks’ fracture toughnesses all increased. Meanwhile, with the rise in the fritting temperature, the sintered Gd_2_O_3_ bulks’ fracture toughnesses increased. The Gd_2_O_3_ bulk possessed the minimum fracture toughness of 10.91 MPa·m^0.5^ when it was sintered at 1400 °C for 6 h, which was 19.5% less than that of the one sintered at 1400 °C for 24 h, with its value of 13.50 MPa·m^0.5^. The Gd_2_O_3_ bulk possessed the fracture toughness of 12.75 MPa·m^0.5^ when it was sintered at 1500 °C for 6 h, which was 8.5% less than that of the one sintered at 1500 °C for 24 h with its value of 14.03 MPa·m^0.5^. The Gd_2_O_3_ bulks possessed the fracture toughnesses of 14.97 MPa·m^0.5^, 15.02 MPa·m^0.5^ and 15.03 MPa·m^0.5^, respectively, when they were sintered at 1600 °C for 6 h, 12 h and 24 h. The fracture toughness was almost unchanged, which was attributed to the fully densified state of the Gd_2_O_3_ bulk sintered at 1600 °C. The densified Gd_2_O_3_ bulk had the fracture toughness of 15.03 MPa·m^0.5^.

## 4. Discussion

**Figure 9:**
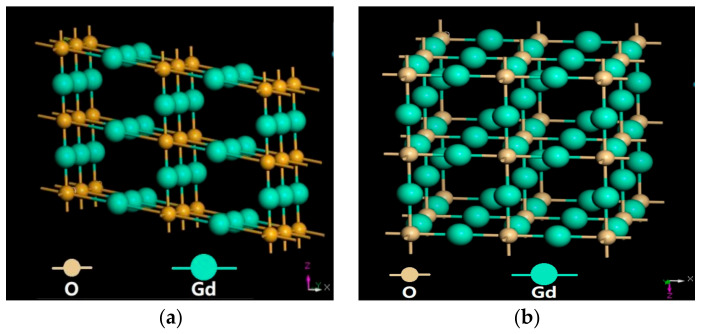
Crystalline structures of the Gd_2_O_3_: (**a**) M-Gd_2_O_3_, (**b**) C-Gd_2_O_3_.

The melting point of Gd_2_O_3_ is 2350 °C, which is very high. Meanwhile, it possesses stable chemical activity, good oxidation resistance and good impact resistance, which makes it suited to serving in high-temperature and harsh circumstance. The crystal structure of TBCs can be modified and become stable by oxide co-doped yttrium oxide-stabilized zirconia. Moreover, Gd possesses a powerful affinity with rest elements and strong chemical stabilities and can be blended into ZrO_2_ cell. Table 7 shows the lattice parameters of the unit cells and the space group of the phases calculated through the CrystalMaker software^®^. M-Gd_2_O_3_ had a monoclinic structure with space group C2/M, as well as the lattice parameters of a (14.095 nm), b (3.5765 nm), c (8.7692 nm) and _β = 100.08°. C-Gd_2_O_3_ had a cubic structure with space group IA-3, and the lattice parameters of a (10.813 nm), b (10.813 nm) and c (10.813 nm) and α = β = γ = 90°. The crystalline structures of monoclinic and cubic Gd_2_O_3_ were shown in Figure 9. Cubic Gd_2_O_3_ had a large lattice constant, which was helpful for enhancing affinity with other elements. Therefore, Gd_2_O_3_ can be added to zirconia as a stabilizer to promote the material’s comprehensive properties used in TBCs.

In this work, Gd_2_O_3_ bulks were sintered at 1400 °C,1500 °C,1600 °C for 6 h to 24 h to study the Gd_2_O_3_ bulks’ microstructure and properties to aid with wider the application of the materials in TBCs. The sintered Gd_2_O_3_ bulks were composed of monoclinic and cubic structures. Meanwhile, the cubic structure took about 90%. The main structure of the sintered Gd_2_O_3_ was cubic. The investigated mechanical properties included hardness, elastic modulus and fracture toughness. The thermal physical properties included the thermal expansion coefficient and thermal conductivity. It was found that the densification of the Gd_2_O_3_ bulk reached 96.16% after 24 h sintering at 1600 °C. When the sintering temperature rose and sintering time extended, the elastic modulus, hardness, fracture toughness and thermal conductivity of the sintered bulks increased, and the CTE decreased gradually. Gd_2_O_3_ bulk possessed the greatest elastic modulus, hardness and fracture toughness of 201.15 GPa, 9.13 GPa and 15.03 MPa·m^0.5^, respectively when it was sintered at 1600 °C for 24 h. Meanwhile, it had the highest thermal conductivity and the lowest CTE of 2.75 W/(m·k) and 6.69 × 10^−6^/°C at 1100 °C. At present, the properties of the co-doped zirconia ceramics used in TBCs are shown in Table 8. The ZrO_2_ bulk exhibited 12.0 GPa hardness, 210 GPa elastic modulus, a fracture toughness of 6 cMPa·m^0.5^, 3.0 W/(m·k) (at room temperature (RT)) thermal conductivity and 9 × 10^−6^/K (at 1100 °C) linear expansion coefficient, respectively [30,31]. The optimized 8YSZ bulk exhibited 13 GPa hardness, 230 GPa elastic modulus, a fracture toughness of 5.1 MPa·m^0.5^, 1.85 W/(m·k) (at 1000 °C) thermal conductivity and 10 × 10^−6^/K (at 1100 °C) CTE, respectively [31,32,33,34]. The 6GdSZ bulk stabilized by Gd_2_O_3_ had 10 GPa hardness, 200 GPa elastic modulus, 1.5 W/(m·k) (at 1100 °C) thermal conductivity and 11.5 × 10^−6^/K (at 1100°) CTE, respectively [35,36]. The 15 wt.% Gd_2_O_3_-GYYZO bulk sintered at 1650 °C for 24 h possessed the hardness, elastic modulus, and fracture toughness, thermal conductivity and thermal expansion coefficient of 15.61 GPa, 306.88 GPa, 7.822 MPa·m^0.5^, 1.04 W/(m·k) and 7.89 × 10^−6^/°C (at 1100 °C), respectively [26]. The increase in the bulk density with the increase in sintering temperature could be attributed to the decrease in pores or voids inside or among the Gd_2_O_3_ powders. It could be assumed that the average grain size of the sintered sample increased moderately with the increase in sintering temperature [22]. For the effect of Gd_2_O_3_ and Yb_2_O_3_ co-doping on the sintering of 8YSZ, Gd_2_O_3_-Yb_2_O_3_-YSZ exhibited better sintering resistance than 8YSZ [37]. It was thought to be the case that sintering process was controlled by diffusion. On the one hand, the shrinkage of Gd_2_O_3_-Yb_2_O_3_-YSZ was lower than that of 8YSZ due to the incorporation of larger and heavier atoms in YSZ. On the other hand, the co-dopants could promote the formation of defect clusters in zirconia crystals. Gd_2_O_3_ will take great effect in adjusting the zirconia’s microstructure and properties to meet the serving environments. The properties of the sintered Gd_2_O_3_ bulk lay the foundation for the properties of Gd_2_O_3_-doped oxide ceramics and can adjust the performances of TBCs prepared with Gd_2_O_3_-doped oxide ceramics further.

## 5. Conclusions

(1) The Gd_2_O_3_ bulk became denser and denser when sintering temperature rose and sintering time extended. The densification of the Gd_2_O_3_ bulk reached 96.16% after sintering at 1600 °C for 24 h, which reached a near-fully dense state.
(2) There was no phase transformation during sintering of the preformed Gd_2_O_3_ bulks. All of the sintered Gd_2_O_3_ bulks were composed of about 90% cubic and 10% monoclinic structures.
(3) When sintering temperature rose and sintering time extended, the hardness, elastic modulus, fracture toughness and thermal conductivity of the sintered Gd_2_O_3_ bulk increased and the CTE decreased gradually. Gd_2_O_3_ bulk sintered at 1600 °C for 24 h possessed the maximum elastic modulus, hardness, fracture toughness and thermal conductivity of 201.15 GPa, 9.13 GPa, 15.03 MPa·m^0.5^ and 2.75 W/(m·k) (at 1100 °C), and the minimum CTE of 6.69 × 10^−6^/°C (at 1100 °C).

## Figures and Tables

**Table 1:** Ball-milling parameters of the powders.

Processing	Parameters
Powders	Gd_2_O_3_
Ball-to-powder ratio in weight	10:1
Grinding ball	Agate ball
Wetting agent	sodium stearate (1 wt.%)
Rotation rate	120 revolutions per minute
Time	20 h

**Table 2:** Parameters of isostatic press and sintering.

Processing	Parameters
Pressure	200 MPa
Keeping time	5 min
Sinter temperature	1600 °C, 1500 °C, 1400 °C
Sinter times	6, 12, 24 h

**Table 3:** The sintered bulks’ porosities analyzed through image processing.

Bulks	1400 °C	1500 °C	1600 °C
6 h	17.9%	7.3%	0.9%
12 h	8.6%	3.8%	0.4%
24 h	4.5%	2.5%	0.2%

**Table 4:** The sintered Gd_2_O_3_ bulks’ densities.

Bulks	1400 °C (g/cm^3^)	1500 °C (g/cm^3^)	1600 °C (g/cm^3^)
6 h	6.086	6.867	7.334
12 h	6.765	7.128	7.377
24 h	7.071	7.228	7.394

**Table 5:** The Gd_2_O_3_ bulks’ densifications.

Bulks	1400 °C	1500 °C	1600 °C
6 h	79.15%	89.21%	95.38%
12 h	87.98%	92.71%	95.94%
24 h	91.96%	94.01%	96.16%

**Table 6:** The sintered Gd_2_O_3_ bulks’ fracture toughnesses/MPa·m^0.5^.

Bulks	1400 °C	1500 °C	1600 °C
6 h	10.91 ± 1.21	12.75 ± 1.54	14.97 ± 1.32
12 h	12.34 ± 1.33	13.84 ± 1.42	15.02 ± 1.43
24 h	13.50 ± 1.28	14.03 ± 1.37	15.03 ± 1.56

**Table 7:** Lattice parameters of the unit cells and the space group of the monoclinic and cubic Gd_2_O_3_.

Crystal Phase	Lattice (Å)	Space Group	Wykoff Coordinates	Angle
**M-Gd_2_O_3_**	a = 14.095 b = 3.5765 c = 8.7692	C2/M	Gd (0.25,0.25,0) O (0,0,0) O (0.5,0,0)	α = γ = 90° β = 100.08°
**C-Gd_2_O_3_**	a = b = c = 10.813	IA-3	Gd (0.25,0,0) O (0.50,0.50,0.50)	α = β = γ = 90°

**Table 8:** Properties of Gd_2_O_3_ and co-doped zirconia ceramics used in TBCs.

Bulks	Hardness (GPa)	Elastic Modulus (GPa)	Fracture Toughness (MPa·m^0.5^)	Thermal Conductivity W/(m·k)	Thermal Expansion Coefficient (at 1100 °C)
Gd_2_O_3_	9.13	201.15	15.03	2.75 (at 1100 °C)	6.69 × 10^−6^/°C
ZrO_2_	12.0 [31]	210 [31]	6.0 [31]	3.0 (at RT) [30]	9 × 10^−6^/K [30]
8YSZ	13 [31]	230 [32]	5.1 [33]	1.85 (at 1000 °C) [16]	10 × 10^−6^/K [34]
6GdSZ	10 [35]	200 [35]	——	1.5 (at 1100 °C) [36]	11.5 × 10^−6^/K [36]
15 wt%Gd_2_O_3_-GYYZO	15.61 [26]	306.88 [26]	7.822 [26]	1.04 (at 1100 °C) [26]	7.89 × 10^−6^/K [26]

## Data Availability

Not applicable.
